# Tumors of the Mouth

**Published:** 1887-03

**Authors:** C. R. Butler


### THE DENTAL REGISTER.

VOL. XLI.] MARCH, 1887. [No. 3.

### COMMUNICATONS.

### Tumors of the Mouth.

BY C. R. BUTLER, M.D., D.D.S.

(Read before the Cleveland Dental Society.)

Tumors of the mouth and their cause are so multifarious that, if we should ever take up their nomination with the briefest analysis possible, we would far exceed the time allotted for the presentation of subjects here. The text-books will give you more than twenty differentiations.

And, notwithstanding the seeming magnitude of the subject, we ought to have such a knowledge of the affections of the oral cavity that we might, with some degree of certainty, determine whether the lesions are benign, or of a malignant character, so that no charge of incompetency could be laid at our door, even if we do not choose to take the cases for treatment.

I shall give you a few cases that have came under my hands the past two years. Dr. W., one of the leading physicians and surgeons of the city, came with the right side of face much tumefied, and the general complexion was bad. Through the hurry and drive of practice, and not suffering much pain, he had allowed the matter to run until considerable general prostration forced him to have his own case attended to, and, on examination, I was satisfied that the antrum was involved, from the heavy sensation complained of when he stooped forward. Advised the removal of the second molar, which was loose, and having no occlusion below. No pus followed the extraction ; but with little force in the alveolus, I made a free opening into the floor of the antrum, when a quantity of very offensive pus came out. After washing it out with warm water, charged with oil of eucalyptis, he took charge of the further treatment. I had an opportunity to see the case, and it was some weeks before it recovered, for the bone in the territory was somewhat involved, the removal of which was hastened by applying aromatic sulphuric acid. A pulpless first bicuspid, that was filled some years before, was doubtless the exciting cause of the inflammation, with the depressed tone of the whole system.

Miss A. B. was induced, through a friend, to consult me. (She was wearing artificial teeth); had been suffering for two or three years with a tumor situated in the left superior canine fossa, and extended forward past the mesial line of the maxillæ, and back to the site of the first molar. Had consulted two dentists and two physicians; the last one in this city, who advised that she go to the hospital and have a part of the jaw removed. This, with the inability to endure longer the pressure of the plate (which had been notched out,) alarmed her. On examination, I found pus exuding from three sinuses, and it looked like a bad case. With an exploring needle, the bone yielded under the tumor, and I struck what proved to be a part of the left superior cuspid. The parts being very tender and highly inflamed, I instituted palliative treatment for a few days, then cut down and removed the broken root and some of the dead bone, the bone being disturbed for so long a time, the root was laying longitudinally. I dressed with a glycerine solution of iodine until the inflammation subsided; then used the acid sulphuric aromatic to dissolve the remaining dead bone.

The case made a good, but slow recovery. The general tone when I first saw her was considerably below par.

MRS. H. B., AGED SIXTY OR MORE.

A tumor situated along the external portion of the superior maxillary, extending from the cite of the second bicuspid from right to left. The woman had been wearing artificial teeth for years, with considerable annoyance at times, with pain and swelling along front of jaw. Before coming into my hands, pus had been discharging to the right. Several attempts had been made to extract a broken root of the right superior central incisor, but without discovering the major cause of the tumor. On making an examination, I told her there was something more than the broken root, for with the exploring needle I struck enamel, indicating imbedded teeth, but she averred that all the teeth had been extracted years ago.

I made an incision through the soft parts, and, with a slender-beaked forcep, removed the two cuspids which had never erupted. They both pointed toward the mesial line, with crown partly outside the bone. They were full size and fair structure. No after treatment required. Another wonderful case of third dentition.

MR. W., AGED SIXTY.

A tumor of the hæmatoma type, situated inside the lip, and low down, close by the left inferior first molar, extending forward to the first bicuspid. I had seen the case more than a year previous to the time of operating, and advised its removal, but as it gave but little trouble in eating, he demurred. Finally it enlarged so as to be in the way in closing of the teeth. It was of a dark purple, or mottled, somewhat irregular and quite firm; largest portion presenting forward.

I first injected a strong solution of chloride of zinc. The second day there was a hole through it crosswise, as the basal portion was much thinner than the body of the tumor. I applied two ligatures, and in two days the mass came away, leaving a clean surface, and, notwithstanding the seeming minor character, its removal produced some shock to the system, for he was of a highly nervous temperament, but it passed off in a few days, and all was well, with no recurrence.

You will observe that I have only briefly alluded to antral troubles in connection with tumors of the mouth, from the fact that it has not been my fortune to have but very few such cases present during my professional life, so I only present such cases as have come under my hands for treatment.

The maxillary sinus is not so subject to disease as some writers might lead you to infer, but when any lesion is established there, we need not fear the making an opening into the cavity for treatment, if the trouble cannot be easily reduced otherwise.

And this gives me an opportunitv to say a few words in favor of making a note of cases that have any peculiar aspect, because, as operators we have, especially in large cities, a great variety in class and condition of people that go to make up the practice of the individual operator, affording an opportunity, if improved, for the aggregation of valuable matter in a short time that would astonish even the close student, and at the same time develop a power of pen-picturing that so many sadly lack that would like to be classed as professional men.

LAST.

Will mention one case more of a " hypertrophic character," that came for treatment in 1884. It was situated under the lip, almost the entire circuit of the superior maxillary; the outer edge was round and very dense, and nearly a quarter of an inch thick. Its removal was accomplished by grasping it with forceps, the lip being held up by an assistant. It was dissected off with scissors. The hemorrhage was not great. The base was somewhat wider than the outer portion. It was dressed by placing a roll of lint, smeared with carbolated vaseline, placed under the lip. The woman had worn a badly adjusted set of upper teeth, but was unable to wear a plate for some time, on account of this cord-like tumor, previous to the operation. I have never seen or heard of a duplicate.

